# Molecular Regulation of NK Cell Maturation

**DOI:** 10.3389/fimmu.2020.01945

**Published:** 2020-08-11

**Authors:** Jiacheng Bi, Xuefu Wang

**Affiliations:** ^1^CAS Key Laboratory of Quantitative Engineering Biology, Shenzhen Institute of Synthetic Biology, Shenzhen Institutes of Advanced Technology, Chinese Academy of Sciences, Shenzhen, China; ^2^Anhui Provincial Laboratory of Inflammatory and Immunity Disease, School of Pharmacy, Anhui Institute of Innovative Drugs, Anhui Medical University, Hefei, China

**Keywords:** NK cells, maturation, development, cytokines, transcriptional regulation

## Abstract

Natural killer (NK) cells are innate lymphocytes specialized in immune surveillance against tumors and infections. To reach their optimal functional status, NK cells must undergo a process of maturation from immature to mature NK cells. Genetically modified mice, as well as *in vivo* and *in vitro* NK cell differentiation assays, have begun to reveal the landscape of the regulatory network involved in NK cell maturation, in which a balance of cytokine signaling pathways leads to an optimal coordination of transcription factor activity. An increased understanding of NK cell maturation will greatly promote the development and application of NK cell-based clinical therapy. Thus, in this review, we summarize the dynamics of NK cell maturation, describe recently identified factors involved in the regulation of the NK cell maturation process, including cytokines and transcription factors, and discuss the importance of NK cell maturation in health and disease.

## Introduction

Natural killer (NK) cells are innate lymphocytes that play an important role in immune surveillance against tumors and virus-infected cells, and are an emerging target for tumor immunotherapy. NK cell immune surveillance is mediated by direct effector functions (such as IFN-γ production and cytotoxicity), as well as immune-regulatory functions [such as interactions with dendritic cells (1–3)] of NK cells. NK cells develop from precursors generated from hematopoietic stem cells (HSCs) in the bone marrow, and then begin the process of maturation before they egress to the periphery for immune surveillance. Since the initial discovery of NK cells in 1975, enormous progress has been made in our understanding of NK cell development and maturation, as well as in identifying the cytokines and transcription factors that are important for these processes. NK cells that reach maturation represent the optimal functional status of the NK cell population at steady-state, and also represent the optimal functional status at the single-cell level, as evidenced by the upregulation of genes encoding cytotoxicity-related effector molecules during NK cell maturation. Compromised NK-dependent immune surveillance usually accompanies impaired NK cell maturation (4, 5). Therefore, it is important to understand the molecular regulatory mechanisms underlying NK cell maturation, which could potentially be exploited for the development of novel therapeutic strategies. In this review, we aim to provide a framework of the current knowledge of NK cell maturation and the factors that regulate this process, including transcription factors and cytokines, and to discuss the importance of NK cell maturation in health and disease.

## An Overview of NK Cell Maturation

### NK Cell Maturation in Mice

**FIGURE 1:**
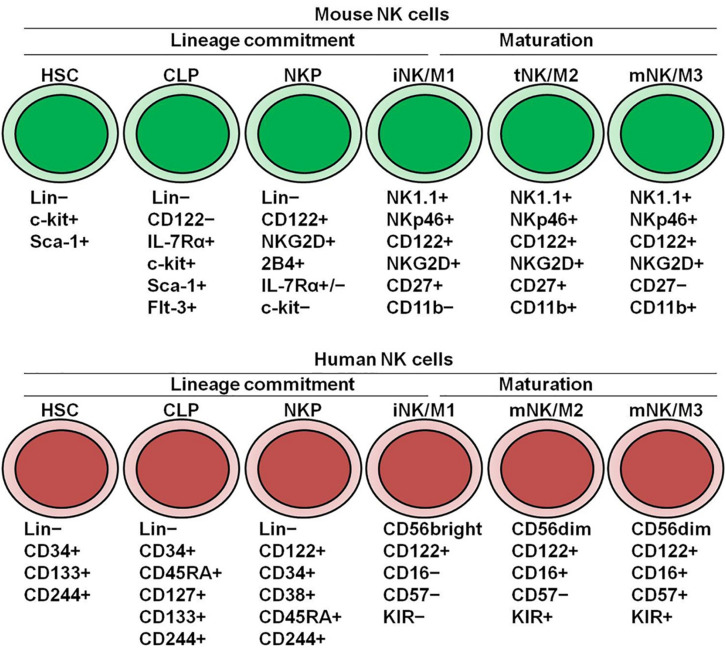
Dynamics of NK cell development and maturation in mice and human. Hematopoietic stem cells (HSCs) differentiate into common lymphoid progenitors (CLPs), and then differentiate into NK cell progenitors (NKPs). The acquisition of CD122 marks NK cell lineage commitment from HSCs. iNKs generated from NKPs continue to mature in the bone marrow and periphery for fully functional acquisition. In mice, CD27/CD11b divides NK cell maturation into three stages. In human, CD56/CD57 divides NK cell maturation into three stages.

In adult mice, NK cells begin their development in the bone marrow, and go through a stepwise cell differentiation process, including HSCs, common lymphoid progenitors (CLPs), preNK cell progenitors (preNKPs), NK cell precursors (NKPs), immature NK cells (iNKs), and mature NK cells (mNKs) (Figure 1). CLPs are Lin^–^IL-7Rα^+^c-Kit^+^SCA-1^+^FLT-3^+^ and can generate pro-B and pre-T cells, as well as the earliest common innate lymphoid cell (ILC) precursor (CILCP). PreNKPs, representing an intermediate stage between CLPs and NKPs, are Lin^–^CD244^+^c-Kit^low^IL-7Rα^+^FLT-3^–^CD122^–^ and comprise both early NK-committed precursors and IL. NKPs, also called refined NKPs (rNKPs), are Lin^–^NK1.1^–^DX5^–^IL-7Rα^+^CD122^+^NKG2D^+^ and differentiate into NK cells (6, 7). Before the final NK lineage commitment, NKPs progress to an iNK stage, which is characterized by the acquisition of NK1.1 and natural cytotoxicity receptor (NCR) expression. However, NK cells at the iNK stage are not yet functionally mature, and their maturation continues in the bone marrow and the periphery. The acquisition of DX5 and Ly49 expression marks the maturation of NK cells (8). To acquire optimized capacity, NK cells continue a late maturation program, which is accompanied by an increase in their effector function and changes in the expression of phenotype markers, such as upregulation of CD11b, CD43, and KLRG1, or downregulation of CD27. Therefore, based on the expression levels of CD27/CD11b, NK cell maturation can be divided into CD27^+^CD11b^–^, CD27^+^CD11b^+^, and CD27^–^CD11b^+^ stages (8). NK cells mature from the CD27^+^CD11b^–^iNK or M1 stage, and then progress to the CD27^+^CD11b^+^ transitional NK cell (tNK) or M2 stage, and finally to the CD27^–^CD11b^+^ terminally mNK or M3 stage (9). During maturation, NK cells gradually increase their cytotoxic capacity but decrease their potential for homeostatic expansion (9).

### NK Cell Maturation in Humans

Human NK cells have developmental trajectories similar to those of mice (Figure 1). HSCs differentiate into CLPs, and then NKPs (10). In humans, the expression of CD122 on NKPs is critical for NK cell lineage commitment. The appearance of CD56 represents the final step in the differentiation of NKPs into NK cells. The maturation of human NK cells can be divided into a CD56^*bright*^ stage and a CD56^*dim*^ stage based on the expression levels of CD56. CD56^*bright*^ NK cells are thought to be immature, and can differentiate into CD56^*dim*^ NK cells with the acquisition of CD16 (11). While two subsets produce inflammatory cytokines, CD56^*dim*^ NK cells have more potent cytolytic activity. CD56^*dim*^ NK cells can further progress into late-maturation stages, with changes in their surface markers and function (12). The terminal maturation of CD56^*dim*^ NK cells with highest cytolytic activity can be defined by the expression of CD57. Approximately 30–60% of all CD56^*dim*^ NK cells in healthy adults express CD57 on their surface (13). Interestingly, high-dimensional, single-cell analysis can identify the high similarity between mouse CD27^–^CD11b^+^ NK cells and human CD56^*dim*^ NK cells and between mouse CD27^+^CD11b^–^ NK cells and human CD56^*bright*^ NK cells (11). Additionally, Fu et al. has showed that CD27 and CD11b can reflect distinct populations of human NK cells from different tissues, functionally similar with their counterparts in mice (14).

Similar to the differentiation process of other innate lymphocytes (15), the maturation of NK cells includes multiple physiological processes. To attain an optimal NK cell population size, the maturation process usually requires the optimal egress of NK cells from the bone marrow, and a finely tuned balance between survival, proliferation, and apoptosis at the steady-state. Meanwhile, optimal NK cell functional status at the single-cell level requires a dedicated transcriptional program dictated by an optimal level of transcriptional factor activity.

### Models Used for Investigation of NK Cell Maturation

Based on the above parameters, several systems are available to investigate the factors involved in the regulation of NK cell maturation:

(1) Knockout mouse models provide a powerful tool to determine the effects of a gene-of-interest on NK cell maturation. Importantly, an increasing number of studies have employed NK cell-specific conditional knockout mouse models, in which Cre recombination-directed gene deletion occurs soon after the acquisition of NKp46 (5, 16–19). This model allows gene deletion that is restricted to NK cells and group 1 innate lymphoid cells (ILC1s) (16); importantly, it also allows the dissection of stage-dependent effects elicited by the gene-of-interest on NK cell maturation.
(2) Adoptive transfer of NK cells into immune-deficient (e.g., *Il2rg*^–/–^*Rag2*^–/–^) mice or reconstitution of the bone marrow in lethally irradiated mice can recapitulate the maturation of NK cells under physiological conditions (20, 21). Different groups of NK cells can be monitored in a competitive manner to assess cell-intrinsic effects through the use of congenic markers such as CD45.1/2.
(3) *In vitro* NK cell differentiation assays using OP9 stromal cells provide an *in vitro* model to mimic cytokine-driven physiological NK cell differentiation from NK precursors (22, 23); this model also allows the determination of cell-specific effects associated with a gene-of-interest.

Several factors and pathways that play a role in NK cell maturation have been identified using the above-mentioned approaches. The results have demonstrated that NK cell maturation is dependent on several critical signaling pathways, and is triggered by a balance between extracellular signals (cytokines) and dictated by an optimal coordination of transcription factor activity. Although NK cell maturation has been extensively studied in mice, knowledge about the factors that control human NK cell maturation remains limited. Nevertheless, advances in gene editing, humanized mice models, single-cell sequencing, mass cytometry, and genome-wide association studies have led to a deeper understanding of how NK cell maturation is regulated in humans.

## Cytokines that Regulate NK Cell Maturation

Increasing evidence suggests that multiple cytokines are involved in NK cell development (Table 1). For instance, IL-7, SCF, and FLT3L are critical for CD122^+^ NKP generation from HSCs, while IL-15 is essential for NK cell lineage commitment and maturation from CD122^+^ NKPs to mNK cells. Additionally, multiple cytokines have been found to be involved in NK cell maturation by modulating IL-15 signaling.

**TABLE 1:** Factors involved in NK cell maturation.

**Factors**	**Effect (Stages affected)**	**Note**	**References**
**Transcription factors**			
E4BP4	+ (CLP, NKP)	Required for IL-15 responsiveness	(54, 55, 57)
TCF-1	− (NKP, iNK, mNK)	Essential for development but limits maturation	(59)
ETS1	+ (NKP, iNK, mNK)	Induce expression of T-bet, GATA3, Blimp1 and ID2	(62, 64)
STAT5	+ (NKP, iNK, mNK)	Downstream of IL-15 receptor	(16, 66–69)
ID2	+ (NKP, iNK, mNK)	Suppress E-box genes; suppress SOCS3	(70–72)
T-bet	+ (iNK, mNK)	Promote S1pr5, IFNG; repress Eomes	(4, 5, 21, 73, 74)
Eomes	+ (iNK, mNK)	Repress T-bet	(21)
TOX1/2	+ (iNK, mNK)	Promotes T-bet expression	(75–77)
PRDM1	+ (iNK, mNK)	induced by T-bet	(22, 78)
Zeb2	+ (iNK, mNK)	Induced by T-bet; Zeb2 KO phenocopy T-bet KO	(5)
GATA3	+ (iNK, mNK)	Dispensable for early NK cell development	(79–84)
SMAD4	+ (iNK, mNK)	TGF-β -independent	(19)
Foxo1	± (iNK)	Promote autophagy (+) or suppress T-bet (−)	(17, 85)
**Cytokines**			
IL-7, SCF, FLT-3L	+ (NKP)	Critical for NKP generation from HSC	(24–32)
Lymphotoxin	+ (NKP)	Essential for early-stage NK development	(33–35)
IL-15	+ (NKP, iNK, mNK)	Promote NK development, maturation, activation, survival and homeostasis	(36–45)
IL-17	− (iNK, mNK)	Induce SOCS3	(47)
TGF-β	− (NKP, iNK, mNK)	Cell cycle arrest before mature stage	(49–53)

*This table lists transcriptional factors or cytokines that either promote (“+”) or suppress (“−”) NK cell maturation, the stages affected (CLP, NKP, iNK, or mNK), as well as the functions or features of these molecules.*

### IL-7, SCF, and FLT-3L

Stroma-free culture has shown that pre-culture in IL-7, SCF, and FLT3L is necessary for NK cell development by inducing IL-15 responsiveness in progenitors (24). CLPs normally exhibit high levels of IL-7 receptor, SCF receptor and FLT-3L receptor; however, the expression of these receptors is gradually lost during the process of NK cell maturation. Nevertheless, these cytokines have distinct roles in NK cell development. For instance, NK cell maturation is almost normal in IL-7Rα- or IL-7-deficient mice (25, 26), whereas the homeostasis of thymus-derived CD127^+^ NK cells is exclusively dependent on IL-7, showing characteristics similar to those of human CD56^*high*^CD16^–^ NK cells (27). IL-7 promotes the survival of CD56^*bright*^ NK cells by increasing BCL2 expression, although it does not increase NK cell cytotoxicity, interferon-gamma (IFN-γ) production, or the expression of activation markers (28). IL-7 alone is not sufficient to support human NK cell development, as evidenced by the findings in human IL-7 knock-in NOD scid gamma (NSG) mice (29). SCF promotes the survival of peripheral c-Kit^+^ NK cells and the absence of c-Kit signaling reduces the generation of NK cells from fetal liver precursors (30). Additionally, a marked deficiency of NK cells has been observed in the spleen of mice lacking FLT3L (31). FLT3L not only potently induces IL-15 responsiveness in progenitors, but can also significantly expand NK cells by increasing the number of IL-15-expressing CD11c^*hi*^ dendritic cells (DCs) (32), indicating that FLT3L is important for the early differentiation of NK cells and NK cell expansion.

### Lymphotoxin

Lymphotoxins alpha (LTα) and beta (LTβ) belong to the tumor necrosis factor (TNF) ligand superfamily. LTα bound to the LTβ receptor (LTβR) constitutes a membrane-bound LTα1β2 heterotrimer that is essential for secondary lymphoid tissue organogenesis, as evidenced by defective lymph node development and altered splenic micro-architecture in *Lta*^–/–^, *Ltb*^–/–^, and *Ltbr*^–/–^ mice. LTα and LTβ are expressed in activated lymphocytes, including T, B, and NK cells, whereas LTβR is expressed exclusively in non-lymphoid tissues, including in bone marrow stromal cells. The ablation of LTα or LTβR leads to impaired early-stage development of NK cells (33–35). Bone marrow stromal cells from *Lta*^–/–^ or *Ltbr*^–/–^ mice cannot efficiently support early NK cell progenitors (35). IL-15 can overcome the arrest of *Lta*^–/–^NK cell development *in vitro* (33); however, NK cell progenitors from wild-type mice generated more NK1.1^+^ cells than NK cell progenitors from *Lta*^–/–^ mice in the presence of IL-15 (35). These findings suggest that the interaction between LTα on NK precursors and LTβR on stromal cells creates a permissive microenvironment that is essential for the early-stage development of NK cells.

### IL-15

IL-15 belongs to the γc family of cytokines, sharing a γc chain with IL-2, -4, -7, -9, and -21. IL-15 signals through IL-15Rβ/γc (encoded by *IL2RG*) heterodimers, either alone with intermediate affinity, or as a complex with IL-15Rα with high affinity (36, 37). A phenotype with a near-complete loss of NK cells is observed in the absence of either IL-15Rα, IL-15Rβ, γc chain, or IL-15 (38–40). In contrast, mice deficient for IL-2, -4, or -7 have normal NK cell development and maturation. During NK cell development, IL-15 acts from CD122^+^NKPs until the terminal maturation of NK cells. Indeed, the ablation of IL-15 does not affect the production of pre-NKPs or NKPs. Although IL-15 is widely expressed in various tissues and by many cell types, IRF-1-dependent IL-15 production in the bone marrow microenvironment is critical for NK cell generation (41). Importantly, the effects of IL-15 on NK cell development and maturation require *trans-*presentation of IL-15 by IL-15α on bone marrow-derived DCs (42–44) in an IL-15α-dose-dependent manner (45). In addition, IL-15 transgenic mice or mice that overexpress IL-15 have a dramatic increase in the number of mNK cells (46). IL-15 is also needed to support mNK cell survival and promote mNK cell expansion in the steady-state. Similar with the observations in mice, IL-15 determines human NK cell maturation and homeostasis, which is supported by evidence from patients with γc mutation and humanized mice, as well as by experiments involving NK cell *ex vivo* development.

### IL-17

IL-17A is a pro-inflammatory cytokine that also plays an immunosuppressive role in some settings (such as in tumors) by recruiting myeloid-derived suppressor cells, promoting angiogenesis, or suppressing CD8^+^ T cells. In addition, our group recently found that IL-17 signaling negatively regulates NK cell maturation and function. IFN-γ production and the cytolytic activity of NK cells, as well as NK cell antitumor and antiviral immune activity, are enhanced in *Il17a*^–/–^mice (47). Although total NK cell numbers are comparable with those in wild-type mice, increased percentages of terminal mature CD27^–^CD11b^+^ NK cells were observed in *Il17a*^–/–^, *Il17f*^–/–^, *Il17a*^–/–^*Il17f*^–/–^, and *Il17ra*^–/–^ mice. Overexpression of IL-17A *in vivo* reduces CD27^–^CD11b^+^ NK cell subsets (47). Mechanistically, IL-17A signaling suppresses IL-15-induced phosphorylation of STAT5 *via* the upregulation of SOCS-3 in NK cells, leading to inhibition of NK cell terminal maturation (47). On the other hand, IL-17A exerts context-dependent effects on NK cells. IL-17 signaling is required for IFN-γ production and the cytolytic activity of NK cells derived from lipopolysaccharide (LPS)-primed mice, as well as for optimal production of granulocyte-macrophage colony-stimulating factor (GM-CSF) by NK cells in fungal infection, which plays a non-redundant role in activating neutrophils for fungal control (48).

### TGF-β

Transforming growth factor beta (TGF-β) is a major immunosuppressive cytokine. Many cell types express TGF-β, and nearly all lymphocyte populations express the TGF-β receptor (TGFβR). Deletion of the TGF-β receptor subunit TGFβ RII enhances mTOR activity and the cytotoxic activity of NK cells in response to IL-15 (49). In the steady-state, NK-specific deletion of TGFβRII in *Ncr1*^*Cre/+*^*; Tgfbr2*^*fl/fl*^ mice has a minimal effect on conventional NK cell maturation and homeostasis (49). In contrast, constitutive TGF-β signaling arrests NK cell maturation (49). Moreover, TGF-β blocks IL-15-induced mTOR activation, thereby suppressing the activation and functions of NK cells (49). Consistent with the observations in mice, treatment with TGF-β *in vitro* inhibits cytokine-stimulated metabolism of human NK cells *via* canonical TGF-β signaling (50). In contrast, transgenic mice expressing a dominant-negative form of TGFβRII under the control of the CD11c promoter (CD11c^*dnTGF*β^ ^*RII*^) exhibit an increase in the number of mature NK cells in the periphery (51). In an *in vitro* assay for the differentiation of NK cell progenitors to less mature CD122^+^NK1.1^+^ and more mature NK1.1^+^DX5^+^ NK cells in the presence of IL-15 and OP9 stromal cells, the addition of TGF-β markedly blocked the derivation of mature NK cells from wild-type precursors, whereas precursors from the CD11c^*dnTGF*β^ ^*RII*^ mice were not affected (51). Mechanistically, TGF-β was found to mediate NK cell immaturity during ontogeny by arresting the cell cycle of NK cells at the least mature CD11b^–^CD43^–^ and intermediate CD11b^+^CD43^–^ stages, as well as by limiting NK cell transition at the terminally mature CD11b^+^CD43^+^ stage (51). As a result of resistance to TGF-β, infant CD11c^*dnTGF*β^ ^*RII*^ mice are protected against murine cytomegalovirus (MCMV) infection (51). In addition, SMAD3 has been found to suppress E4BP4-mediated NK cell development and effector functions, as evidenced by the increased number of mNK cells in *Smad3*^–/–^ mice and the increased levels of granzyme B, IL-2, and IFN-γ in *Smad3*^–/–^NK cells (52). Meanwhile, the silencing of *Smad3* in the human NK-92 cell line allows for the upregulation of E4BP4, which subsequently promotes IFN-γ production (53). The differences in phenotypes between CD11c^+^ cells and NKp46^+^ cells following blockade of TGFβR signaling might be due to the abrogation of the TGFβR signal at different stages of NK cell development/maturation. Further studies are required to reveal the temporal regulation of NK cell maturation by TGF-β.

## Transcription Factors that Regulate NK Cell Maturation

**FIGURE 2:**
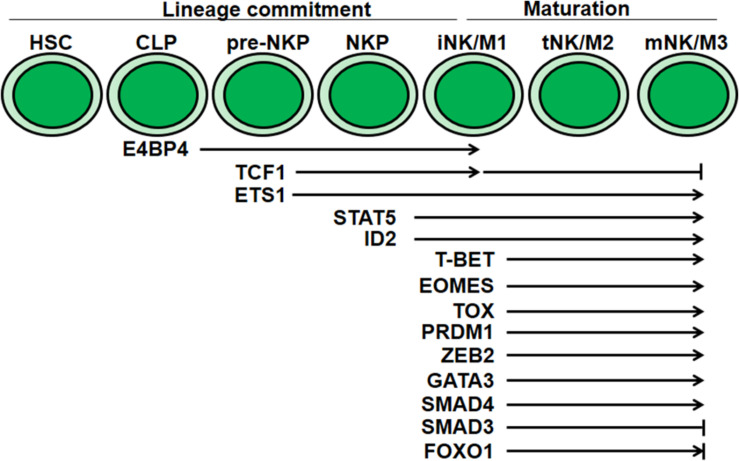
Transcriptional regulation of NK cell development and maturation. Multiple transcriptional factors mediate regulation at distinct stages during NK cell development and maturation. E4BP4, TCF1 and ID2 are essential for NK cell lineage commitment. T-BET, EOMES, TOX, PRDM1, GATA3, ZEB2, and SMAD4 are critical for NK cell maturation. The discrepancy of FOXO1 in NK cell maturation needs further confirmation.

Natural killer cell development is also regulated by sequential and coordinated transcription factor activity (Figure 2). For instance, transcription factors such as E4BP4, TOX, and ID2 are required for NK cell lineage commitment, while ID2, T-bet, Eomes, and ZEB2 are required for NK cell maturation. Transcription factors that mediate NK cell maturation usually promote the expression of genes coding for effector molecules, receptors responsible for egress, and cell-surface maturation markers.

### E4BP4

E4BP4 is expressed in CLPs (54). *E4bp4*^–/–^ mice specifically lack NK cells (55). Indeed, the numbers of pre-NKPs, rNKPs, iNK cells, and mNK cells, but not those of CLPs, are reduced in the bone marrow of *E4bp4*^–/–^ mice. Ectopic expression of E4BP4 in *E4bp4*^–/–^CLPs can rescue NK cell production (54). These findings indicate that E4BP4 acts at the CLP stage and is required for NK cell lineage commitment. Pre-NKPs and rNKPs are not affected in the absence of IL-15 signaling; however, the absence of E4BP4 results in the failure of IL-15-responsive NKP production. Specifically, IL-15 cannot rescue NK cell production in *E4bp4*^–/–^ bone marrow (55). In contrast, ectopic expression of E4BP4 enables limited NK cell production, even in the absence of IL-15 signaling (55). However, ectopic expression of Eomes, ID2, or T-bet cannot rescue NK cell production in the absence of E4BP4. Furthermore, E4BP4 was found to directly regulate the expression of Eomes and ID2 (54), while the histone H2A deubiquitinase MYSM1 has been found to be critical for the recruitment of E4BP4 to the *ID2* locus and be required for NK cell maturation, but not NK lineage commitment (23). In addition, *NOTCH1* has been identified as an E4BP4 target gene in NK cells (56). The abrogation of Notch signaling blocked NK cell production, similar to that observed in *E4bp4*^–/–^mice. Exposure to Notch peptide ligands at an early stage rescued the defective NK cell development from *E4bp4*^–/–^ progenitors (56). Moreover, SUMOylation and phosphorylation can regulate E4BP4 activity and then influence NK cell development (56). The ablation of either SUMOylation or phosphorylation sites significantly increased the transcriptional activity of E4BP4 and promoted the production of NK cells. Therefore, not only the expression level but also the activity of E4BP4 is critical for NK cell development. However, the regulation of E4BP4 activity by post-translational modifications needs further investigation. Firth et al. reported that specific ablation of E4BP4 in either iNK or mNK cells had no effect on NK cell lineage maintenance and homeostasis (57). Therefore, these findings confirm that E4BP4 is required for NK lineage commitment, but not for the maturation from the iNK to the mNK stage or the survival or maintenance of mNK cells. Although it has been reported that the upregulation of E4BP4 can enhance IFN-γ production in NK-92 cells (53), the roles of E4BP4 in human NK lineage commitment and development are not well defined.

### TCF1

TCF1 (encoded by Tcf7 gene) is a member of the high-mobility group (HMG) of proteins, and is important for T cell development and function. TCF1 was initially found to bind to the *Ly49A* promoter and be required for the acquisition of Ly49A expression during NK cell development (58). Loss of TCF1 results in a greater than 50% reduction in the levels of LY49D on NK cells (59). Subsequently, studies on a TCF1 reporter mouse strain demonstrated that TCF1 was expressed by pre-NKPs, NKPs, iNK cells, and mNK cells, but not CLPs, in the bone marrow (60). Pre-NKPs, NKPs, and mNK cell numbers were reduced in *Tcf7*^–/–^ mice. Moreover, detailed analysis in splenic NK cells showed that TCF1 expression was high in CD27^+^CD11b^–^ NK cells, declined with NK cell maturation, and disappeared in CD27^–^CD11b^+^ NK cells. Surprisingly, the number of terminally mature NK cells in *Tcf7*^–/–^ mice is significantly increased. These findings indicate that TCF1 is essential for NK cell development, but limits NK cell terminal maturation. In humans, TCF1 has been found to be uniquely expressed in circulating CD56^*bright*^ NK cells and not in CD56^*dim*^ NK cells (61). The effects of TCF1 on human NK cell development and maturation, as well as the underlying mechanisms, need further investigation.

### ETS1

ETS1 is the founding member of the ETS family of winged helix-turn-helix transcription factors and is highly evolutionarily conserved. ETS1 has been shown to function from the pre-NKP stage and is required for the expression of various transcription factors, including T-bet and ID2, activating NKRs, including NKp46, Ly49D, and Ly49H, and signaling molecules (62). The levels of mNK cells are significantly decreased in the bone marrow and spleen of *Ets1*^–/–^mice (62). Moreover, *Ets1*^–/–^ mNK cells display impaired effector function and decreased expression of activating NKRs, but increased expression of inhibitory NKRs (62, 63). Meanwhile, in human NK cells, Taveirne et al. revealed that ETS1 induced the expression of transcription factors such as T-bet, GATA3, and BLIMP-1 that determine NK cell development, suggesting that ETS1 might play a similar role as in humans (64). Moreover, IL-2 and IL-15 can increase ETS1 expression through ERK1/2 signaling in human NK cells (65). These observations indicate that ETS1 is essential for NK cell development. However, the stage-specific requirement of ETS1 for NK cell maturation requires further investigation.

### STAT5

Downstream of IL-15 receptor, JAK-1 associates with IL-2Rβ, leading to STAT3 phosphorylation, while JAK-3 associates with IL-2Rγc, resulting in STAT5 phosphorylation (66). The JAK-1/3–STAT5 pathway has been identified as being essential for NK cell maturation. Germ-line deletion of *Jak1* or *Stat5* and conditional deletion of *Jak1* or *Stat5* in NKp46^+^ cells both lead to a marked reduction in NK cell levels, as well as inhibition of NK cell maturation in the bone marrow and the periphery (16, 67). Consistent with the reduced levels of mNK cells, *Jak1*^*flox/flox*^*Ncr1*-iCre Tg or *Stat5*^*flox/flox*^*Ncr1*-iCre Tg mice showed defective NK cell-dependent tumor surveillance. Moreover, STAT5 tetramers are required for the maturation of NK cells from the iNK to the mNK state in bone marrow and spleen, as evidenced by the substantial decrease in mNK cell numbers, but normal levels of NKPs and iNK cells, in STAT5A-STAT5B tetramer-deficient double knock-in mice (68). STAT5b mutation also leads to defective human NK cell maturation and impaired lytic function (69). These observations indicate that JAK-1/3-STAT5 is critical for NK cell maturation.

### ID2

ID2 is a member of the inhibitor of DNA-binding (ID) protein family, which acts by preventing E-proteins, such as E2A, E2-2, and HEB, from binding E-box (CANNTG)-containing target genes through heterodimerization. ID2 is expressed at high levels after NK cell lineage commitment and in all subsequent stages (70). ID2 was first reported to play an essential role in the generation of peripheral lymphoid organs and NK cells through the use of *Id2*^–/–^ mice (71). Although *Id2* deficiency does not reduce Lin^–^CD122^+^ NK cell progenitors in the bone marrow, it does lead to reduced NK cell numbers and impaired maturation of NK cells, as evidenced by the decreased expression of CD43 and CD11b maturation markers, as well as granzyme expression (72). A more recent study showed that the hematopoietic deletion and acute deletion of ID2, or ID2 deletion in NKp46^+^ cells, results in the loss of most NK cells (70). This indicates that ID2 is required for NK cell maintenance at all stages of development. ID2 suppresses E-box genes required for NK cell maturation, suppresses SOCS-3 expression, and is required for normal IL-15 receptor signaling. Strong IL-15 receptor stimulation by IL-2 pre-ligated to an anti-IL-2 antibody, or SOCS-3 deficiency, partially restores homeostasis in *Id2*-deficient NK cells.

In support of the role of ID2 in NK cell maturation, deficiency of MYSM1, which mediates the recruitment of E4BP4 to the *Id2* locus, leads to defects in NK cell maturation, but not NK lineage specification or commitment (23). MYSM1 also plays a role in maintaining an open chromatin structure at the *Id2* locus.

### T-Bet and Eomes

T-bet (T-box expressed in T cells, encoded by *TBX21*) is a member of the T-box family of transcription factors that are primarily expressed by T cells and NK cells. In *Tbet*^–/–^ mice, the number of NK cells is significantly decreased in the spleen, liver, and peripheral blood, but modestly increased in bone marrow NK cells (4). These defects result from the impaired egress of NK cells from the bone marrow. T-bet directly regulates S1pr5 by binding to its locus (73), and loss of T-bet leads to the decreased expression of S1pr5 (74). In addition, T-bet-deficient NK cells express lower levels of the maturation markers CD43, CD11b, and DX5, and higher levels of c-Kit and integrin alpha-v, which reflects the immaturity of NK cells (4). Therefore, T-bet is essential for NK cell maturation.

Another T-box transcription factor, Eomes (eomesodermin), also plays non-redundant roles in NK cell maturation. In mice, most spleen NK cells express Eomes. Expression of Eomes marks NK cell maturation, and Eomes^+^ NK cells are characterized by the expression of DX5 and the absence of TRAIL expression (21). Eomes is required for NK cell maturation. Hematopoietic deletion of Eomes in *Eomes*^*flox/flox*^*Vav*-Cre^+^ mice results in a substantial reduction in the number of NK cells in the spleen and blood, and to a lesser extent in the liver, lymph nodes, and bone marrow. Loss of Eomes also renders NK cells phenotypically similar to Eomes^–^ NK cells derived from wild-type mice, with expression of TRAIL but lacking DX5, as well as decreased levels of Ly49 receptors and CD11b. In addition, TRAIL^+^ iNKs require Eomes for conversion to DX5^+^ mature NK cells following their transfer into *Il2rg*^–/–^*Rag2*^–/–^ mice. Eomes is also required to maintain NK cell maturity, as temporal deletion of Eomes in mature NK cells results in the loss of the maturation marker DX5 and upregulation of TRAIL.

Despite the critical roles of Eomes and T-bet in NK cell maturation, loss of either transcription factor does not markedly impair the effector functions of NK cells at the single-cell level *in vitro*, further confirming that these factors have roles in NK cell maturation, but not in NK cell identity.

### TOX

The TOX (thymocyte selection-associated HMG box) protein belongs to a family of evolutionarily conserved DNA-binding proteins. Besides TOX, family members include TOX2, TOX3, and TOX4. The TOX protein is highly expressed in iNK and mNK cells in the bone marrow (75). The loss of TOX significantly reduces the frequency and number of mNK cells in the periphery and bone marrow, with a severe block in the transition from the iNK to mNK stage and, to a lesser extent, from the NKP to the iNK stage. ID2 expression is significantly reduced in the NK cells that remain in *Tox*^–/–^mice; however, ID2 expression does not rescue the defective maturation of *Tox*^–/–^NK cells. In addition, Yun et al. demonstrated that TOX enhances the maturation of human NK cells, and affects, though not directly regulates, T-bet expression during NK cell maturation (76). This indicates that TOX is required for NK cell maturation both in humans and in mice. However, the regulatory circuit downstream of TOX during NK cell maturation requires further investigations. Besides TOX, TOX2 has been found to play a critical role in human NK cell maturation. Vong et al. found that TOX2 is preferentially expressed in human NK cells among several immune cell populations and is upregulated during human NK cell maturation. TOX2 is essential for human NK cell maturation and cytotoxicity. TOX2 directly upregulates T-bet expression, and T-bet overexpression can rescue the TOX2 knockdown-mediated NK cell maturation defects (77). These results imply that TOX2 functions upstream of ID2 in human NK cells. However, the role of TOX2 in murine NK cell maturation remains to be elucidated. Combined, these results show TOX and TOX2 play a crucial role in NK cell maturation.

### PRDM1

The expression of the transcriptional repressor B lymphocyte-induced maturation protein 1 (Blimp-1, also known as PRDM1) can be induced by IL-15 stimulation (22), and its expression is T-bet-dependent (78). Blimp-1 is constitutively expressed by NK cells and is upregulated during NK cell maturation in both humans and mice. Loss of Blimp-1 affects NK cell homeostasis, leading to increased NK cell numbers in the bone marrow and lymph nodes and reduced NK cell numbers in the liver and the lungs. Loss of Blimp-1 also leads to impaired NK cell maturation, as shown by the decreased levels of the CD27^–^CD11b^+^ NK cell subset and expression of the maturation markers CD43 and KLRG1. However, Blimp-1 is dispensable for most effector functions of murine NK cells, and even suppresses the production of IFN-γ, TNF, and LTαin human NK cells by directly binding to multiple conserved regulatory regions (78). An impaired response to IL-15 in the absence of Blimp-1 might be the cause of the aberrant maturation of Blimp-1^–/–^ NK cells.

### Zeb2

T-bet also synergizes with ZEB2 to promote NK cell maturation. T-bet is necessary and sufficient to induce ZEB2 expression, and ZEB2 expression positively correlates with NK cell maturation (5). ZEB2 is required for normal NK cell homeostasis. Deletion of ZEB2 in NK cells results in the retention of NK cells in the bone marrow, and reduced NK cell numbers in the periphery. ZEB2 overexpression leads to a reduced number of NK cells in the bone marrow. Moreover, mice with NK-specific *Zeb2* deletion lack mature CD27^–^ NK cells, while ZEB2 overexpression increases the numbers of mature CD27^–^ NK cells. The reduced numbers of mature NK cells might be due to poor survival and response to IL-15, as well as the downregulation of S1pr5 expression in ZEB2-deficient NK cells. Additionally, the levels of the maturation markers KLRG1 and CD146 are also proportional to the levels of ZEB2 expression. This implies that ZEB2 promotes NK cell maturation. Interestingly, although the absence of ZEB2 in NK cells compromises the overall NK cell-mediated immune response, as evidenced by the increased susceptibility of NK-*Zeb2*^–/–^ mice to B16F10 lung metastasis, NK cell effector functions at the single-cell level *in vitro* are mostly preserved, or even increased.

ZEB2-deficient NK cells phenocopy T-bet-deficient NK cells in terms of reduced numbers of mature NK cells, higher rates of apoptosis, impaired egress from the bone marrow, and the expression of a series of surface molecules (5). Impaired maturation of ZEB2- or T-bet-deficient NK cells is not further aggravated in ZEB2/T-bet double-deficient NK cells. Furthermore, overexpression of ZEB2 partially rescues the defects of T-bet-deficient NK cells related to cell numbers, mature NK cell levels, expression of maturation markers such as KLRG1, and surveillance against tumor metastasis.

### GATA3

GATA3 belongs to a family of six transcription factors termed GATA binding proteins (79). GATA3 is expressed in the hematopoietic system and throughout the NK lineage, from NKPs to iNK and mNK cells (80). Deletion of GATA3 in all hematopoietic cells at the early stage of development does not result in the loss of conventional NK cells, indicating that GATA3 is dispensable for early NK cell development (81–83). However, *NKp46*-Cre-*Gata3*^*flox/flox*^ mice show a decrease in NK cell numbers in the spleen and liver, and an increase in the bone marrow (84). The maturation markers CD11b and KLRG1 are also expressed at lower levels on NK cells in mice with either hematopoietic or *NKp46* promoter-mediated deletion of *Gata*3 (81, 84). In addition, although NK cell cytolytic activity is preserved in the absence of GATA3, both from the early stage of development and in the immature NK cell stage, IFN-γ production by NK cells is impaired in *NKp46*-Cre-*Gata3*^*flox/flox*^ mice. Accordingly, control of early Listeria infection is compromised in mice lacking GATA3. This indicates that GATA3 is required for NK cell maturation.

### SMAD3 and SMAD4

The binding of TGF-β to its tetrameric receptor phosphorylates the transcription factors Smad2/3, which then binds to Smad4 to regulate the expression of target genes. Consistently, Smad3 has been also found to suppress NK cell E4BP4-mediated development and effector functions, as evidenced by the increased number of mNK cells in *Smad3*^–/–^ mice and the increased levels of granzyme B, IL-2,and IFN-γ in *Smad3*^–/–^ NK cells (52). Meanwhile, the silencing of *Smad3* was found to result in the upregulation of E4BP4, leading to the subsequent promotion of IFN-γ production in human NK-92 cells (53). SMAD4 generally acts as a mediator of the TGF-β signaling pathway; however, a recent study found that SMAD4 promotes NK cell homeostasis and maturation in a TGF-β-independent manner (19). SMAD4 deficiency in NK cells compromises NK cell granzyme B expression, as well as NK cell antitumor and antiviral immunity. SMAD4 cooperates with JUNB to transactivate *Gzmb* gene expression. SMAD4 is also required to maintain NK cell homeostasis, possibly by promoting c-Kit expression. SMAD4 deficiency impairs NK cell maturation, as evidenced by the decreased percentage of KLRG1^+^ cells and CD27^–^CD11b^+^ mature subsets among NK cells, possibly due to the decreased expression of Blimp-1 in these NK cells. Interestingly, these effects of SMAD4 on NK cells are independent of TGF-β signaling, as SMAD4 deficiency also results in decreased KLRG1^+^ and CD27^–^CD11b^+^ NK cells, and in decreased expression of the *Gzmb* gene in a Tgfbr2- deficient background.

### FOXO1

To date, forkhead box O1 (FOXO1) is the only transcription factor reported to negatively regulate NK cell maturation. FOXO1 is abundantly expressed in iNKs and its expression subsequently decreases during the NK cell maturation process (17), opposite to that observed for T-bet. FOXO1 is normally localized in the nucleus; however, following cellular activation (e.g., by stimulatory cytokines), it is phosphorylated and translocates to the cytoplasm. In the nucleus, FOXO1 directly represses *Tbx21* transcription. Accordingly, conditional FOXO1 deletion in NKp46^+^ cells promotes NK cell maturation and effector functions. However, it has also been reported that FOXO1 interacts with ATG7 to induce autophagy, which is important for immature NK cell viability, and that conditional deletion of FOXO1 in NKp46^+^ cells compromises NK cell development and MCMV clearance (85). These observations highlight the complexity associated with the activity of this molecule.

## Defective NK Cell Maturation and Disease

Natural killer cells play critical roles in immune surveillance against viral infections and cancers. They can be directly activated without priming and have the potential to kill infected or transformed cells. However, the compromised functional competence of NK cells can lead to immune escape of viruses and cancer cells. NK cell maturation is critical for the acquisition of full functional competence. Consequently, defective maturation of NK cells is associated with chronic viral infection and cancer progression.

### Defective NK Cell Maturation and Infection

Natural killer cells play critical roles in defending against viral infection. Evidence indicates that hosts without circulating NK cells suffer multiple severe viral infections, including varicella-zoster virus (VZV), cytomegalovirus (CMV), Epstein–Barr virus (EBV), and herpes simplex virus (HSV), and have a higher risk of death from these infections (86). Mutations in *GATA2* might result in NK cell deficiency (87). The highest expression of GATA2 was found in the CD56^*bright*^ subset. CD56^*bright*^ NK cells were absent in patients with *GATA2* mutations, indicating that GATA2 is critical for human NK cell development. Further experimental work may provide insights into these patients and the particular mutation to better understand the role of GATA2 in NK cell biology. In addition, patients with MCM4 deficiency have a selective loss of CD56^*dim*^ in NK cells, indicating that MCM4 mutation may interrupt the maturational transition of CD56^*bright*^ NK cells to CD56^*dim*^ NK cells. Notably, mutations in the IL-15 receptor, JAK-3, or STAT5 in humans results in the defective development and maturation of NK cells and increases the risk of viral infection (86). Functional NK cell deficiency resulting from gene mutations in humans also leads to increased susceptibility to multiple infections. For instance, IL-21 promotes the functional maturation of both murine and human NK cells (88, 89). The loss-of-function of human IL-21 or its receptor leads to impaired NK cell cytotoxicity and recurrent infections (90, 91). Meanwhile, viral chronic infection, such as that associated with hepatitis B virus (HBV), hepatitis C virus (HCV), human immunodeficiency virus (HIV), and severe acute respiratory syndrome coronavirus 2 (SARS-CoV-2), results in NK cell depletion or dysfunction, characterized by reduced numbers, proliferation, activity, and expression of activating receptors, as well as increased apoptosis and expression of inhibitory receptors (92, 93). Defective maturation of NK cells affects the outcomes of viral infections. For instance, the frequency of the CD11b^+^CD27^–^NK subset is higher in *Il1r8*^–/–^ mice compared with intact ones. Enhanced NK cell maturation in *Il1r8*^–/–^ mice promotes NK-cell-mediated resistance to CMV infection (94). Together, these results indicate that NK cells are critical for host defense against viral infections and NK cell maturation is required for the acquisition of their full functional competence.

### Defective NK Cell Maturation and Cancer

Cancer immune surveillance by NK cells contributes to the exploration and application of NK cells in clinical cancer treatment. Hosts with dysregulated NK cells have an increased cancer risk (95). NK cell defects have been observed in a variety of patients with different types of advanced cancer, and contribute to defective immunity against cancer progression (96). Immunotherapy based on NK cells has displayed robust anticancer efficacy in preclinical and clinical trials (97). There is some evidence to show that NK cell maturation is also blocked during tumor growth. It has been demonstrated that multiple lineages of tumors, including breast cancer, colon cancer, and melanoma cell lines, interrupt NK cell functional maturation and impair the antitumor capacity of NK cells (98). The maturation defects in these cancer hosts are associated with a significant reduction in the number of IL-15Rα^+^ cells in the bone marrow, and these defects can be rescued by overexpression of IL-15 (98). Interestingly, it has been reported that GSK3 kinase inhibition in human NK cells that were expanded with IL-15 enhances CD57 acquisition and late-stage maturation, which is accompanied by higher TNF and IFN-γ production, elevated natural cytotoxicity, and increased antibody-dependent cellular cytotoxicity (99). Furthermore, the adoptive transfer of NK cells expanded in the presence of a GSK3 kinase inhibitor led to a stronger and more durable antitumor effect in a xenogeneic ovarian cancer model. Mechanistically, GSK3 kinase inhibition increases the expression of T-bet, ZEB2, and BLIMP-1, transcription factors that are associated with late-stage NK cell maturation. This demonstrates that NK cell functional maturation is required for the efficacy of NK cell-based cancer immunotherapy.

## Perspectives

As discussed above, NK cell maturation is a process coordinated by a network that consists of transcription factors, signaling pathways, and cytokines. Through the use of stage-specific, gene-modified mouse models, as well as other complementary systems, additional molecules that play a role in the NK cell maturation process are being identified, further expanding our knowledge of this basic topic in NK cell biology.

On the other hand, NK cells are composed of different subsets with different maturation status. For example, mouse NK cells with a self-MHC-specific Ly49 inhibitory receptor (e.g., the Ly49I/C^+^ NK cell subset) are tolerant toward self-MHC –expressing cells, but are better producers of IFN-γ in response to cross-linking of activation receptors (100). These better-educated Ly49I/C^+^ NK cells display a more mature phenotype, compared with Ly49I/C^–^ NK cells (9). Another example is that NK cells can be divided into two distinct subsets: CD49a^+^ and CD49a^–^. Different from conventional CD49a- NK cells, CD49a^+^ NK cells are tissue resident. Tissue-resident NK (trNK) cells have been observed in diverse organs and tissues, including thymus, uterus, skin, adipose, liver, and salivary. The transcriptional factors that instruct the development of trNK cells are different from those of cNK cells. For instance, mice with *E4bp4* deficiency lack conventional NK (cNK) cells, but have trNK cells in liver and uterus (101). In addition, the phenotype markers and necessary factors for the development of trNK cells have been found to display notable tissue-specific properties. For example, the development of trNK cells in the thymus with CD127^+^CD49b^+^CD49a^–^ phenotype requires IL-7 and GATA3 (27). The origin, developmental process, transcriptional factor, and functional competence of trNK cells in mice and human remain less-studied, though some recent studies have started shedding insights into the comparison between the cNK and trNK (102, 103). Interestingly, CD49a^+^ liver-resident NK cells display an immature phenotype, compared with CD49a^–^ conventional NK cells. The impacts of maturation status on the differentiation programs and specific features (such as liver-residency of liver-resident NK cells) of these NK cell subsets still remains to be investigated.

Another conceptual breakthrough in NK cell biology relates to the immunological memory and adaption of NK cells (104, 105). Increasing evidence indicates that NK cells recall haptens, viruses, and cytokines (106). For antigen-specific NK cell memory responses in a murine model of hapten-induced contact hypersensitivity (CHS), NK cells require CXCR6 expression for such process (107). Also, NK cell-mediated CHS is dependent on IL-12, IFN-α, and IFN-γ (108). For generation of protective memory NK cell response during MCMV infection, pro-inflammatory cytokines (e.g., IL-12 and downstream STAT4) (109) and co-stimulatory receptors (e.g., CD226) (110) play crucial roles. Moreover, in order to form NK cell memory, NK cells require Zbtb32 to undergo a proliferative burst and clonal expansion in response to infection (111), require BNIP3 and BNIP3L -dependent clearance of damaged mitochondria by autophagy (112), and require pro-apoptotic factor Bim for contraction (113). Considering the above-discussed effects of NK cell maturation on NK cell effector functions, the question arises as to whether adaptive NK cell memory also depends on NK cell maturation status. An appropriate model of NK cell memory should be employed that reflects the relationship between an immunological memory and maturation of NK cells (114). Although molecular mechanisms on the differentiation of adaptive NK cells remains to be fully revealed, the immunological memory of NK cells support the potential for NK cells in promoting better clinical outcomes during disease treatment.

More efforts are required, however, before knowledge in this area can be exploited for disease treatment. For example, NK cell activity is dysregulated in autoimmune diseases (115), which potentially affects the maturation process in the bone marrow even if the disease is confined to a region away from the bone marrow. However, relatively few studies have investigated the maturation status of NK cells under such conditions. On the other hand, NK cells are functionally exhausted in the tumor microenvironment (116, 117). The levels of CD27^–^CD11b^–^ tumor-infiltrating NK cells are associated with tumor progression in a murine Lewis lung cancer (LLC) model (118), indicating that the tumor microenvironment displays region-specific immune features, and further suggests that NK cell maturation is differentially regulated when compared with the normal condition; however, this idea requires further investigation.

## Author Contributions

JB and XW conceived and wrote the manuscript, and designed the figures. Both authors contributed to the article and approved the submitted version.

## Conflict of Interest

The authors declare that the research was conducted in the absence of any commercial or financial relationships that could be construed as a potential conflict of interest.

## References

[1] Thomas R Yang X. NK-DC Crosstalk in immunity to microbial infection. *J Immunol Res.* (2016) 2016:6374379. 10.1155/2016/6374379 PMC5206438 28097157

[2] Ferlazzo G Morandi B. Cross-talks between natural killer cells and distinct subsets of dendritic cells. *Front Immunol.* (2014) 5:159. 10.3389/fimmu.2014.00159 24782864 PMC3989561

[3] Cooper MA Fehniger TA Fuchs A Colonna M Caligiuri MA. NK cell and DC interactions. *Trends Immunol.* (2004) 25:47–52. 14698284 10.1016/j.it.2003.10.012

[4] Townsend MJ Weinmann AS Matsuda JL Salomon R Farnham PJ Biron CA T-bet regulates the terminal maturation and homeostasis of NK and Valpha14i NKT cells. *Immunity.* (2004) 20:477–94. 15084276 10.1016/s1074-7613(04)00076-7

[5] van Helden MJ Goossens S Daussy C Mathieu AL Faure F Marcais A Terminal NK cell maturation is controlled by concerted actions of T-bet and Zeb2 and is essential for melanoma rejection. *J Exp Med.* (2015) 212:2015–25. 26503444 10.1084/jem.20150809 PMC4647267

[6] Geiger TL Sun JC. Development and maturation of natural killer cells. *Curr Opin Immunol.* (2016) 39:82–9. 26845614 10.1016/j.coi.2016.01.007 PMC4801705

[7] Colucci F Caligiuri MA Di Santo JP. What does it take to make a natural killer? *Nat Rev Immunol.* (2003) 3:413–25. 12766763 10.1038/nri1088

[8] Kim S Iizuka K Kang HS Dokun A French AR Greco S In vivo developmental stages in murine natural killer cell maturation. *Nat Immunol.* (2002) 3:523–8. 12006976 10.1038/ni796

[9] Chiossone L Chaix J Fuseri N Roth C Vivier E Walzer T. Maturation of mouse NK cells is a 4-stage developmental program. *Blood.* (2009) 113:5488–96. 19234143 10.1182/blood-2008-10-187179

[10] Di Vito C Mikulak J Mavilio D. On the way to become a natural killer cell. *Front Immunol.* (2019) 10:1812. 10.3389/fimmu.2019.01812 31428098 PMC6688484

[11] Crinier A Milpied P Escaliere B Piperoglou C Galluso J Balsamo A High-dimensional single-cell analysis identifies organ-specific signatures and conserved NK cell subsets in humans and mice. *Immunity.* (2018) 49:971–86.e975. 30413361 10.1016/j.immuni.2018.09.009 PMC6269138

[12] Bjorkstrom NK Riese P Heuts F Andersson S Fauriat C Ivarsson MA Expression patterns of NKG2A, KIR, and CD57 define a process of CD56dim NK-cell differentiation uncoupled from NK-cell education. *Blood.* (2010) 116:3853–64. 20696944 10.1182/blood-2010-04-281675

[13] Lopez-Verges S Milush JM Pandey S York VA Arakawa-Hoyt J Pircher H CD57 defines a functionally distinct population of mature NK cells in the human CD56dimCD16+ NK-cell subset. *Blood.* (2010) 116:3865–74. 20733159 10.1182/blood-2010-04-282301 PMC2981540

[14] Fu B Wang F Sun R Ling B Tian Z Wei H. CD11b and CD27 reflect distinct population and functional specialization in human natural killer cells. *Immunology.* (2011) 133:350–9. 21506999 10.1111/j.1365-2567.2011.03446.x PMC3112344

[15] Kansler ER Li MO. Innate lymphocytes-lineage, localization and timing of differentiation. *Cell Mol Immunol.* (2019) 16:627–33. 30804475 10.1038/s41423-019-0211-7 PMC6804950

[16] Eckelhart E Warsch W Zebedin E Simma O Stoiber D Kolbe T A novel Ncr1-Cre mouse reveals the essential role of STAT5 for NK-cell survival and development. *Blood.* (2011) 117:1565–73. 21127177 10.1182/blood-2010-06-291633

[17] Deng Y Kerdiles Y Chu J Yuan S Wang Y Chen X Transcription factor Foxo1 is a negative regulator of natural killer cell maturation and function. *Immunity.* (2015) 42:457–70. 25769609 10.1016/j.immuni.2015.02.006 PMC4400836

[18] Wang F Meng M Mo B Yang Y Ji Y Huang P Crosstalks between mTORC1 and mTORC2 variagate cytokine signaling to control NK maturation and effector function. *Nat Commun.* (2018) 9:4874. 10.1038/s41467-018-07277-9 PMC6242843 30451838

[19] Wang Y Chu J Yi P Dong W Saultz J Wang Y SMAD4 promotes TGF-beta-independent NK cell homeostasis and maturation and antitumor immunity. *J Clin Invest.* (2018) 128:5123–36. 30183689 10.1172/JCI121227 PMC6205382

[20] Banh C Miah SM Kerr WG Brossay L. Mouse natural killer cell development and maturation are differentially regulated by SHIP-1. *Blood.* (2012) 120:4583–90. 23034281 10.1182/blood-2012-04-425009 PMC3512235

[21] Gordon SM Chaix J Rupp LJ Wu J Madera S Sun JC The transcription factors T-bet and Eomes control key checkpoints of natural killer cell maturation. *Immunity.* (2012) 36:55–67. 22261438 10.1016/j.immuni.2011.11.016 PMC3381976

[22] Kallies A Carotta S Huntington ND Bernard NJ Tarlinton DM Smyth MJ A role for Blimp1 in the transcriptional network controlling natural killer cell maturation. *Blood.* (2011) 117:1869–79. 21131593 10.1182/blood-2010-08-303123

[23] Nandakumar V Chou Y Zang L Huang XF Chen SY. Epigenetic control of natural killer cell maturation by histone H2A deubiquitinase, MYSM1. *Proc Natl Acad Sci USA.* (2013) 110:E3927–36. 24062447 10.1073/pnas.1308888110 PMC3799335

[24] Williams NS Moore TA Schatzle JD Puzanov IJ Sivakumar PV Zlotnik A Generation of lytic natural killer 1.1+, Ly-49- cells from multipotential murine bone marrow progenitors in a stroma-free culture: definition of cytokine requirements and developmental intermediates. *J Exp Med.* (1997) 186:1609–14. 9348320 10.1084/jem.186.9.1609 PMC2199105

[25] He YW Malek TR. Interleukin-7 receptor alpha is essential for the development of gamma delta + T cells, but not natural killer cells. *J Exp Med.* (1996) 184:289–93. 8691145 10.1084/jem.184.1.289 PMC2192680

[26] Moore TA von Freeden-Jeffry U Murray R Zlotnik A. Inhibition of gamma delta T cell development and early thymocyte maturation in IL-7 -/- mice. *J Immunol.* (1996) 157:2366–73. 8805634

[27] Vosshenrich CA Garcia-Ojeda ME Samson-Villeger SI Pasqualetto V Enault L Richard-Le Goff O A thymic pathway of mouse natural killer cell development characterized by expression of GATA-3 and CD127. *Nat Immunol.* (2006) 7:1217–24. 17013389 10.1038/ni1395

[28] Michaud A Dardari R Charrier E Cordeiro P Herblot S Duval M. IL-7 enhances survival of human CD56bright NK cells. *J Immunother.* (2010) 33:382–90. 20386468 10.1097/CJI.0b013e3181cd872d

[29] Matsuda M Ono R Iyoda T Endo T Iwasaki M Tomizawa-Murasawa M Human NK cell development in hIL-7 and hIL-15 knockin NOD/SCID/IL2rgKO mice. *Life Sci Alliance.* (2019) 2:e201800195. 10.26508/lsa.201800195 PMC6445396 30936185

[30] Colucci F Di Santo JP. The receptor tyrosine kinase c-kit provides a critical signal for survival, expansion, and maturation of mouse natural killer cells. *Blood.* (2000) 95:984–91. 10648413

[31] McKenna HJ Stocking KL Miller RE Brasel K De Smedt T Maraskovsky E Mice lacking flt3 ligand have deficient hematopoiesis affecting hematopoietic progenitor cells, dendritic cells, and natural killer cells. *Blood.* (2000) 95:3489–97. 10828034

[32] Williams NS Klem J Puzanov IJ Sivakumar PV Bennett M Kumar V. Differentiation of NK1.1+, Ly49+ NK cells from flt3+ multipotent marrow progenitor cells. *J Immunol.* (1999) 163:2648–56. 10453005

[33] Iizuka K Chaplin DD Wang Y Wu Q Pegg LE Yokoyama WM Requirement for membrane lymphotoxin in natural killer cell development. *Proc Natl Acad Sci USA.* (1999) 96:6336–40. 10339588 10.1073/pnas.96.11.6336 PMC26882

[34] Smyth MJ Johnstone RW Cretney E Haynes NM Sedgwick JD Korner H Multiple deficiencies underlie NK cell inactivity in lymphotoxin-alpha gene-targeted mice. *J Immunol.* (1999) 163:1350–3. 10415034

[35] Wu Q Sun Y Wang J Lin X Wang Y Pegg LE Signal via lymphotoxin-beta R on bone marrow stromal cells is required for an early checkpoint of NK cell development. *J Immunol.* (2001) 166:1684–9. 11160211 10.4049/jimmunol.166.3.1684

[36] Dubois S Mariner J Waldmann TA Tagaya Y. IL-15Ralpha recycles and presents IL-15 In trans to neighboring cells. *Immunity.* (2002) 17:537–47. 12433361 10.1016/s1074-7613(02)00429-6

[37] Fehniger TA Caligiuri MA. Interleukin 15: biology and relevance to human disease. *Blood.* (2001) 97:14–32. 11133738 10.1182/blood.v97.1.14

[38] Lodolce JP Boone DL Chai S Swain RE Dassopoulos T Trettin S IL-15 receptor maintains lymphoid homeostasis by supporting lymphocyte homing and proliferation. *Immunity.* (1998) 9:669–76. 9846488 10.1016/s1074-7613(00)80664-0

[39] Kennedy MK Glaccum M Brown SN Butz EA Viney JL Embers M Reversible defects in natural killer and memory CD8 T cell lineages in interleukin 15-deficient mice. *J Exp Med.* (2000) 191: 771–80. 10704459 10.1084/jem.191.5.771 PMC2195858

[40] Vosshenrich CA Ranson T Samson SI Corcuff E Colucci F Rosmaraki EE Roles for common cytokine receptor gamma-chain-dependent cytokines in the generation, differentiation, and maturation of NK cell precursors and peripheral NK cells in vivo. *J Immunol.* (2005) 174:1213–21. 15661875 10.4049/jimmunol.174.3.1213

[41] Ogasawara K Hida S Azimi N Tagaya Y Sato T Yokochi-Fukuda T Requirement for IRF-1 in the microenvironment supporting development of natural killer cells. *Nature.* (1998) 391:700–3. 9490414 10.1038/35636

[42] Sandau MM Schluns KS Lefrancois L Jameson SC. Cutting edge: transpresentation of IL-15 by bone marrow-derived cells necessitates expression of IL-15 and IL-15R alpha by the same cells. *J Immunol.* (2004) 173:6537–41. 15557143 10.4049/jimmunol.173.11.6537

[43] Burkett PR Koka R Chien M Chai S Boone DL Ma A. Coordinate expression and trans presentation of interleukin (IL)-15Ralpha and IL-15 supports natural killer cell and memory CD8+ T cell homeostasis. *J Exp Med.* (2004) 200:825–34. 15452177 10.1084/jem.20041389 PMC2213280

[44] Schluns KS Nowak EC Cabrera-Hernandez A Puddington L Lefrancois L Aguila HL. Distinct cell types control lymphoid subset development by means of IL-15 and IL-15 receptor alpha expression. *Proc Natl Acad Sci USA.* (2004) 101:5616–21. 15060278 10.1073/pnas.0307442101 PMC397446

[45] Lee GA Liou YH Wang SW Ko KL Jiang ST Liao NS. Different NK cell developmental events require different levels of IL-15 trans-presentation. *J Immunol.* (2011) 187:1212–21. 21715685 10.4049/jimmunol.1100331

[46] Fehniger TA Suzuki K Ponnappan A VanDeusen JB Cooper MA Florea SM Fatal leukemia in interleukin 15 transgenic mice follows early expansions in natural killer and memory phenotype CD8+ T cells. *J Exp Med.* (2001) 193:219–31. 11208862 10.1084/jem.193.2.219 PMC2193336

[47] Wang X Sun R Hao X Lian ZX Wei H Tian Z. IL-17 constrains natural killer cell activity by restraining IL-15-driven cell maturation via SOCS3. *Proc Natl Acad Sci USA.* (2019) 116:17409–18. 31405974 10.1073/pnas.1904125116 PMC6717263

[48] Bar E Whitney PG Moor K Reis e Sousa C LeibundGut-Landmann S. IL-17 regulates systemic fungal immunity by controlling the functional competence of NK cells. *Immunity.* (2014) 40:117–27. 24412614 10.1016/j.immuni.2013.12.002

[49] Viel S Marcais A Guimaraes FS Loftus R Rabilloud J Grau M TGF-beta inhibits the activation and functions of NK cells by repressing the mTOR pathway. *Sci Signal.* (2016) 9:ra19. 10.1126/scisignal.aad1884 26884601

[50] Zaiatz-Bittencourt V Finlay DK Gardiner CM. Canonical TGF-beta signaling pathway represses human NK Cell metabolism. *J Immunol.* (2018) 200:3934–41. 29720425 10.4049/jimmunol.1701461

[51] Marcoe JP Lim JR Schaubert KL Fodil-Cornu N Matka M McCubbrey AL TGF-beta is responsible for NK cell immaturity during ontogeny and increased susceptibility to infection during mouse infancy. *Nat Immunol.* (2012) 13:843–50. 22863752 10.1038/ni.2388 PMC3426626

[52] Tang PM Zhou S Meng XM Wang QM Li CJ Lian GY Smad3 promotes cancer progression by inhibiting E4BP4-mediated NK cell development. *Nat Commun.* (2017) 8:14677. 10.1038/ncomms14677 PMC5343519 28262747

[53] Wang QM Tang PM Lian GY Li C Li J Huang XR Enhanced cancer immunotherapy with Smad3-silenced NK-92 cells. *Cancer Immunol Res.* (2018) 6:965–77. 29915022 10.1158/2326-6066.CIR-17-0491

[54] Male V Nisoli I Kostrzewski T Allan DS Carlyle JR Lord GM The transcription factor E4bp4/Nfil3 controls commitment to the NK lineage and directly regulates Eomes and Id2 expression. *J Exp Med.* (2014) 211: 635–42. 24663216 10.1084/jem.20132398 PMC3978281

[55] Gascoyne DM Long E Veiga-Fernandes H de Boer J Williams O Seddon B The basic leucine zipper transcription factor E4BP4 is essential for natural killer cell development. *Nat Immunol.* (2009) 10:1118–24. 19749763 10.1038/ni.1787

[56] Kostrzewski T Borg AJ Meng Y Filipovic I Male V Wack A Multiple levels of control determine how E4bp4/Nfil3 regulates NK cell development. *J Immunol.* (2018) 200:1370–81. 29311361 10.4049/jimmunol.1700981 PMC5812440

[57] Firth MA Madera S Beaulieu AM Gasteiger G Castillo EF Schluns KS Nfil3-independent lineage maintenance and antiviral response of natural killer cells. *J Exp Med.* (2013) 210:2981–90. 24277151 10.1084/jem.20130417 PMC3865482

[58] Ioannidis V Kunz B Tanamachi DM Scarpellino L Held W. Initiation and limitation of Ly-49A NK cell receptor acquisition by T cell factor-1. *J Immunol.* (2003) 171:769–75. 12847244 10.4049/jimmunol.171.2.769

[59] Held W Clevers H Grosschedl R. Redundant functions of TCF-1 and LEF-1 during T and NK cell development, but unique role of TCF-1 for Ly49 NK cell receptor acquisition. *Eur J Immunol.* (2003) 33:1393–8. 12731066 10.1002/eji.200323840

[60] Jeevan-Raj B Gehrig J Charmoy M Chennupati V Grandclement C Angelino P The transcription factor Tcf1 contributes to normal NK cell development and function by limiting the expression of granzymes. *Cell Rep.* (2017) 20:613–26. 28723565 10.1016/j.celrep.2017.06.071

[61] Collins PL Cella M Porter SI Li S Gurewitz GL Hong HS Gene regulatory programs conferring phenotypic identities to human NK Cells. *Cell.* (2019) 176:348–60.e312. 30595449 10.1016/j.cell.2018.11.045 PMC6329660

[62] Ramirez K Chandler KJ Spaulding C Zandi S Sigvardsson M Graves BJ Gene deregulation and chronic activation in natural killer cells deficient in the transcription factor ETS1. *Immunity.* (2012) 36:921–32. 22608498 10.1016/j.immuni.2012.04.006 PMC3389314

[63] Barton K Muthusamy N Fischer C Ting CN Walunas TL Lanier LL The Ets-1 transcription factor is required for the development of natural killer cells in mice. *Immunity.* (1998) 9:555–63. 9806641 10.1016/s1074-7613(00)80638-x

[64] Taveirne S Wahlen S Van Loocke W Kiekens L Persyn E Van Ammel E The transcription factor ETS1 is an important regulator of human NK cell development and terminal differentiation. *Blood.* (2020) 136:288–98. 32350509 10.1182/blood.2020005204

[65] Grund EM Spyropoulos DD Watson DK Muise-Helmericks RC. Interleukins 2 and 15 regulate Ets1 expression via ERK1/2 and MNK1 in human natural killer cells. *J Biol Chem.* (2005) 280:4772–8. 15563472 10.1074/jbc.M408356200

[66] Miyazaki T Kawahara A Fujii H Nakagawa Y Minami Y Liu ZJ Functional activation of Jak1 and Jak3 by selective association with IL-2 receptor subunits. *Science.* (1994) 266:1045–7. 7973659 10.1126/science.7973659

[67] Witalisz-Siepracka A Klein K Prinz D Leidenfrost N Schabbauer G Dohnal A Loss of JAK1 drives innate immune deficiency. *Front Immunol.* (2018) 9:3108. 10.3389/fimmu.2018.03108 30671064 PMC6331462

[68] Lin JX Du N Li P Kazemian M Gebregiorgis T Spolski R Critical functions for STAT5 tetramers in the maturation and survival of natural killer cells. *Nat Commun.* (2017) 8:1320. 10.1038/s41467-017-01477-5 PMC5673064 29105654

[69] Vargas-Hernandez A Witalisz-Siepracka A Prchal-Murphy M Klein K Mahapatra S Al-Herz W Human signal transducer and activator of transcription 5b (STAT5b) mutation causes dysregulated human natural killer cell maturation and impaired lytic function. *J Allergy Clin Immunol.* (2020) 145:345–57.e349. 31600547 10.1016/j.jaci.2019.09.016 PMC7155380

[70] Delconte RB Shi W Sathe P Ushiki T Seillet C Minnich M The helix-loop-helix protein ID2 governs NK cell fate by tuning their sensitivity to interleukin-15. *Immunity.* (2016) 44:103–15. 26795246 10.1016/j.immuni.2015.12.007

[71] Yokota Y Mansouri A Mori S Sugawara S Adachi S Nishikawa S Development of peripheral lymphoid organs and natural killer cells depends on the helix-loop-helix inhibitor Id2. *Nature.* (1999) 397:702–6. 10067894 10.1038/17812

[72] Boos MD Yokota Y Eberl G Kee BL. Mature natural killer cell and lymphoid tissue-inducing cell development requires Id2-mediated suppression of E protein activity. *J Exp Med.* (2007) 204:1119–30. 17452521 10.1084/jem.20061959 PMC2118569

[73] Jenne CN Enders A Rivera R Watson SR Bankovich AJ Pereira JP T-bet-dependent S1P5 expression in NK cells promotes egress from lymph nodes and bone marrow. *J Exp Med.* (2009) 206:2469–81. 19808259 10.1084/jem.20090525 PMC2768857

[74] Walzer T Chiossone L Chaix J Calver A Carozzo C Garrigue-Antar L Natural killer cell trafficking in vivo requires a dedicated sphingosine 1-phosphate receptor. *Nat Immunol.* (2007) 8:1337–44. 17965716 10.1038/ni1523

[75] Aliahmad P de la Torre B Kaye J. Shared dependence on the DNA-binding factor TOX for the development of lymphoid tissue-inducer cell and NK cell lineages. *Nat Immunol.* (2010) 11:945–52. 20818394 10.1038/ni.1930 PMC2943551

[76] Yun S Lee SH Yoon SR Kim MS Piao ZH Myung PK TOX regulates the differentiation of human natural killer cells from hematopoietic stem cells in vitro. *Immunol Lett.* (2011) 136: 29–36. 21126536 10.1016/j.imlet.2010.11.008

[77] Vong QP Leung WH Houston J Li Y Rooney B Holladay M TOX2 regulates human natural killer cell development by controlling T-BET expression. *Blood.* (2014) 124:3905–13. 25352127 10.1182/blood-2014-06-582965 PMC4282154

[78] Smith MA Maurin M Cho HI Becknell B Freud AG Yu J PRDM1/Blimp-1 controls effector cytokine production in human NK cells. *J Immunol.* (2010) 185:6058–67. 20944005 10.4049/jimmunol.1001682 PMC3864810

[79] Evans T Reitman M Felsenfeld G. An erythrocyte-specific DNA-binding factor recognizes a regulatory sequence common to all chicken globin genes. *Proc Natl Acad Sci USA.* (1988) 85:5976–80. 3413070 10.1073/pnas.85.16.5976 PMC281888

[80] Rosmaraki EE Douagi I Roth C Colucci F Cumano A Di Santo JP. Identification of committed NK cell progenitors in adult murine bone marrow. *Eur J Immunol.* (2001) 31:1900–9. 11433387 10.1002/1521-4141(200106)31:6<1900::aid-immu1900>3.0.co;2-m

[81] Samson SI Richard O Tavian M Ranson T Vosshenrich CA Colucci F GATA-3 promotes maturation, IFN-gamma production, and liver-specific homing of NK cells. *Immunity.* (2003) 19:701–11. 14614857 10.1016/s1074-7613(03)00294-2

[82] Sojka DK Plougastel-Douglas B Yang L Pak-Wittel MA Artyomov MN Ivanova Y Tissue-resident natural killer (NK) cells are cell lineages distinct from thymic and conventional splenic NK cells. *eLife.* (2014) 3:e01659. 10.7554/eLife.01659 PMC3975579 24714492

[83] Yagi R Zhong C Northrup DL Yu F Bouladoux N Spencer S The transcription factor GATA3 is critical for the development of all IL-7Ralpha-expressing innate lymphoid cells. *Immunity.* (2014) 40:378–88. 24631153 10.1016/j.immuni.2014.01.012 PMC4026797

[84] Ali AK Oh JS Vivier E Busslinger M Lee SH. NK cell-specific Gata3 ablation identifies the maturation program required for bone marrow exit and control of proliferation. *J Immunol.* (2016) 196:1753–67. 26773150 10.4049/jimmunol.1501593

[85] Wang S Xia P Huang G Zhu P Liu J Ye B FoxO1-mediated autophagy is required for NK cell development and innate immunity. *Nat Commun.* (2016) 7:11023. 10.1038/ncomms11023 PMC4820827 27010363

[86] Orange JS. Natural killer cell deficiency. *J Allergy Clin Immunol.* (2013) 132:515–25. 23993353 10.1016/j.jaci.2013.07.020 PMC3917661

[87] Mace EM Hsu AP Monaco-Shawver L Makedonas G Rosen JB Dropulic L Mutations in GATA2 cause human NK cell deficiency with specific loss of the CD56(bright) subset. *Blood.* (2013) 121:2669–77. 23365458 10.1182/blood-2012-09-453969 PMC3617632

[88] Brady J Hayakawa Y Smyth MJ Nutt SL. IL-21 induces the functional maturation of murine NK cells. *J Immunol.* (2004) 172:2048–58. 14764669 10.4049/jimmunol.172.4.2048

[89] Bonanno G Mariotti A Procoli A Corallo M Scambia G Pierelli L Interleukin-21 induces the differentiation of human umbilical cord blood CD34-lineage- cells into pseudomature lytic NK cells. *BMC Immunol.* (2009) 10:46. 10.1186/1471-2172-10-46 19712464 PMC2743656

[90] Kotlarz D Zietara N Uzel G Weidemann T Braun CJ Diestelhorst J Loss-of-function mutations in the IL-21 receptor gene cause a primary immunodeficiency syndrome. *J Exp Med.* (2013) 210:433–43. 23440042 10.1084/jem.20111229 PMC3600901

[91] Kotlarz D Zietara N Milner JD Klein C. Human IL-21 and IL-21R deficiencies: two novel entities of primary immunodeficiency. *Curr Opin Pediatr.* (2014) 26:704–12. 25321844 10.1097/MOP.0000000000000160

[92] Zheng M Gao Y Wang G Song G Liu S Sun D Functional exhaustion of antiviral lymphocytes in COVID-19 patients. *Cell Mol Immunol.* (2020) 17:533–5. 32203188 10.1038/s41423-020-0402-2 PMC7091858

[93] Hammer Q Ruckert T Romagnani C. Natural killer cell specificity for viral infections. *Nat Immunol.* (2018) 19:800–8. 30026479 10.1038/s41590-018-0163-6

[94] Molgora M Bonavita E Ponzetta A Riva F Barbagallo M Jaillon S IL-1R8 is a checkpoint in NK cells regulating anti-tumour and anti-viral activity. *Nature.* (2017) 551:110–4. 29072292 10.1038/nature24293 PMC5768243

[95] Imai K Matsuyama S Miyake S Suga K Nakachi K. Natural cytotoxic activity of peripheral-blood lymphocytes and cancer incidence: an 11-year follow-up study of a general population. *Lancet.* (2000) 356:1795–9. 11117911 10.1016/S0140-6736(00)03231-1

[96] Zhang C Hu Y Shi C. Targeting natural killer cells for tumor immunotherapy. *Front Immunol.* (2020) 11:60. 10.3389/fimmu.2020.00060 32140153 PMC7042203

[97] Sivori S Vacca P Del Zotto G Munari E Mingari MC Moretta L. Human NK cells: surface receptors, inhibitory checkpoints, and translational applications. *Cell Mol Immunol.* (2019) 16:430–41. 30778167 10.1038/s41423-019-0206-4 PMC6474200

[98] Richards JO Chang X Blaser BW Caligiuri MA Zheng P Liu Y. Tumor growth impedes natural-killer-cell maturation in the bone marrow. *Blood.* (2006) 108:246–52. 16556890 10.1182/blood-2005-11-4535 PMC1895835

[99] Cichocki F Valamehr B Bjordahl R Zhang B Rezner B Rogers P GSK3 inhibition drives maturation of NK cells and enhances their antitumor activity. *Cancer Res.* (2017) 77:5664–75. 28790065 10.1158/0008-5472.CAN-17-0799 PMC5645243

[100] Kim S Poursine-Laurent J Truscott SM Lybarger L Song YJ Yang L Licensing of natural killer cells by host major histocompatibility complex class I molecules. *Nature.* (2005) 436:709–13. 16079848 10.1038/nature03847

[101] Sojka DK. Uterine natural killer cell heterogeneity: lessons from mouse models. *Front Immunol.* (2020) 11:290. 10.3389/fimmu.2020.00290 32153593 PMC7046796

[102] Hashemi E Malarkannan S. Tissue-resident NK cells: development, maturation, and clinical relevance. *Cancers.* (2020) 12:1553. 10.3390/cancers12061553 PMC7352973 32545516

[103] Valero-Pacheco N Beaulieu AM. Transcriptional regulation of mouse tissue-resident natural killer cell development. *Front Immunol.* (2020) 11:309. 10.3389/fimmu.2020.00309 32161593 PMC7052387

[104] Wu LS Wang JY. Warm up, cool down, and tearing apart in NK cell memory. *Cell Mol Immunol.* (2018) 15:1095–7. 30487549 10.1038/s41423-018-0188-7 PMC6269542

[105] Wang X Peng H Tian Z. Innate lymphoid cell memory. *Cell Mol Immunol.* (2019) 16:423–9. 30796350 10.1038/s41423-019-0212-6 PMC6474199

[106] Peng H Tian Z. Natural killer cell memory: progress and implications. *Front Immunol.* (2017) 8:1143. 10.3389/fimmu.2017.01143 28955346 PMC5601391

[107] Paust S Gill HS Wang BZ Flynn MP Moseman EA Senman B Critical role for the chemokine receptor CXCR6 in NK cell-mediated antigen-specific memory of haptens and viruses. *Nat Immunol.* (2010) 11:1127–35. 20972432 10.1038/ni.1953 PMC2982944

[108] Majewska-Szczepanik M Paust S von Andrian UH Askenase PW Szczepanik M. Natural killer cell-mediated contact sensitivity develops rapidly and depends on interferon-alpha, interferon-gamma and interleukin-12. *Immunology.* (2013) 140:98–110. 23659714 10.1111/imm.12120 PMC3809710

[109] Sun JC Madera S Bezman NA Beilke JN Kaplan MH Lanier LL. Proinflammatory cytokine signaling required for the generation of natural killer cell memory. *J Exp Med.* (2012) 209:947–54. 22493516 10.1084/jem.20111760 PMC3348098

[110] Nabekura T Kanaya M Shibuya A Fu G Gascoigne NR Lanier LL. Costimulatory molecule DNAM-1 is essential for optimal differentiation of memory natural killer cells during mouse cytomegalovirus infection. *Immunity.* (2014) 40:225–34. 24440149 10.1016/j.immuni.2013.12.011 PMC3943894

[111] Beaulieu AM Zawislak CL Nakayama T Sun JC. The transcription factor Zbtb32 controls the proliferative burst of virus-specific natural killer cells responding to infection. *Nat Immunol.* (2014) 15:546–53. 24747678 10.1038/ni.2876 PMC4404304

[112] O’Sullivan TE Johnson LR Kang HH Sun JC. BNIP3- and BNIP3L-mediated mitophagy promotes the generation of natural killer cell memory. *Immunity.* (2015) 43:331–42. 26253785 10.1016/j.immuni.2015.07.012 PMC5737626

[113] Min-Oo G Bezman NA Madera S Sun JC Lanier LL. Proapoptotic Bim regulates antigen-specific NK cell contraction and the generation of the memory NK cell pool after cytomegalovirus infection. *J Exp Med.* (2014) 211:1289–96. 24958849 10.1084/jem.20132459 PMC4076589

[114] Sun H Sun C Xiao W Sun R. Tissue-resident lymphocytes: from adaptive to innate immunity. *Cell Mol Immunol.* (2019) 16:205–15. 30635650 10.1038/s41423-018-0192-y PMC6460493

[115] Van Kaer L Postoak JL Wang C Yang G Wu L. Innate, innate-like and adaptive lymphocytes in the pathogenesis of MS and EAE. *Cell Mol Immunol.* (2019) 16:531–9. 30874627 10.1038/s41423-019-0221-5 PMC6804597

[116] Bi J Tian Z. NK cell exhaustion. *Front Immunol.* (2017) 8:760. 10.3389/fimmu.2017.00760 28702032 PMC5487399

[117] Huang Q Huang M Meng F Sun R. Activated pancreatic stellate cells inhibit NK cell function in the human pancreatic cancer microenvironment. *Cell Mol Immunol.* (2019) 16:87–9. 29628497 10.1038/s41423-018-0014-2 PMC6318268

[118] Jin J Fu B Mei X Yue T Sun R Tian Z CD11b(-)CD27(-) NK cells are associated with the progression of lung carcinoma. *PLoS One.* (2013) 8:e61024. 10.1371/journal.pone.0061024 23565296 PMC3614924

